# Genetic diversity in enhancer II region of HBV genotype D and its association with advanced liver diseases

**DOI:** 10.1371/journal.pone.0261721

**Published:** 2022-01-04

**Authors:** Majid Khan, Sanaullah Khan, Mehrunnisa Fatima Gondal, Safia Bibi, Bakht Tarin Khan, Abdul Majid, Ayesha Khattak, Muhammad Nasir Khabir, Muhammad Anwar, Aisha Gul, Maryam Naseem, Sobia Attaullah

**Affiliations:** 1 Department of Zoology, University of Peshawar, Peshawar, Khyber Pakhtunkhwa, Pakistan; 2 Rural Health Centre Barki, District Lahore, Pakistan; 3 Department of Zoology, Kohat University of Science and Technology Kohat, Kohat, Khyber Pakhtunkhwa, Pakistan; 4 Department of Zoology, University of Buner, Buner, Khyber Pakhtunkhwa, Pakistan; 5 Department of Zoology, Islamia College Peshawar University, Peshawar, Khyber Pakhtunkhwa, Pakistan; University of Health Sciences Lahore, PAKISTAN

## Abstract

**Background:**

Hepatitis B Virus (HBV) is one of the most common human infectious agents, and the mutations in its genome may cause chronic hepatitis (CH), liver cirrhosis (LC), and hepatocellular carcinoma (HCC). This study was designed to characterize the enhancer-II (Enh-II) region of X gene in HBV positive patients to assess the association of such mutations with CH, LC, and HCC.

**Methods:**

HBV positive samples (N = 200) with patients’ demographic and clinical data were collected from different regions of Khyber Pakhtunkhwa (KP), Pakistan. The Enh-II region of the HBx gene was sequenced and zanalyzed for polymorphism associated with advanced liver disease. Univariate and logistic regression analyses were performed to evaluate potent mutations associated with a risk for LC and HCC.

**Results:**

HBV Enh-II region sequences analysis revealed 25 different mutations. The highest frequency of mutations S101F (62.2%), A102V/R/G/I (56.25%), M103L/A (68.75%)were found in HCC, followed in LC and CH patients as 57.1%, 42.8%, 28.52% 16%, 15.2% and 18.4% respectively. H94 deletion in the α-box of the Enh-II region, associated with a high risk of HCC was found in half of the HCC patients. This deletion was present in 28.5% of LC and 6.5% of CH patients. Importantly, the high frequency of some notable mutations such as E109A/Y, A110S/K, Y111D/E, and F112L was first time reported in the entire study population. The frequencies of these mutations were high in HCC (43.75%, 37.5%, 50% and 43.75% respectively) as compared to LC (14.28%, 14.28%, 28.2% and 42.8%) and CH patients (12.8%, 15.2%, 16.8% and 16% respectively).

**Conclusion:**

Mutations associated with LC and HCC are prevalent in the Enh-II region in Pakistani HBV isolates. The mutations found are alarming in CH patients as these may progress to LC and HCC in a large number of patients.

## Introduction

HBV has been proved to be the most important virological factor for the development of advanced liver disease as LC and HCC. The replication capabilities of the virus and the accumulation of potent mutations in the HBV genome, are common events for HCC development, and thus considered a life-threatening pathogen leading to significant rates of mortality worldwide [1, 2]. According to WHO, 257 million people are living with HBV infection with an estimated number of 887,000 deaths in 2015 attributed to HBV complications [3].

The infectious virion has an internal capsid (core particle) that protects the partially double-stranded DNA genome. HBV genome consists of four overlapping reading frames (ORFs) corresponding to four major genes—polymerase (P), surface (S), core (C), and regulatory (X). Hepatitis B X (HBx) is the smallest of HBV genes, encoding for 154 amino acids that constitute 17.5 kilodalton protein. HBx is involved in transcription regulation and interacts with a variety of cellular pathways responsible for oncogenesis [4]. The HBV genome contains two transcriptional enhancer (Enh) regions i.e. Enh-I and Enh-II, which are important for viral replication. The Enh-II, located in nt 1636–1741, overlaps the HBx coding region and stimulates the transcriptional activity of the surface, core, and HBx gene promoters which shows high hepatocyte-specific activity [5]. Enh-II is further composed of two regions i.e. box-α (nt 1644–1666) and box-β (nt 1702–1713) [6]. Enh-II region of the HBx plays a key role in controlling the transcription of all genes in a hepatocyte-specific manner. Moreover, Enh-II plays an important role in the viral life cycle by regulating the formation of the 3.5 kb pre-genomic RNA which is then translated to produce the viral core and polymerase proteins and HBeAg [7].

Enh-II stimulates the transcription of surface protein-I (SP1), surface protein-II, and X protein in a position and orientation-independent manner [8]. On the other hand, this element regulates the basal core proteins (BCP) region in a position and orientation-dependent manner, functioning as a core upstream regulatory sequence, which overlaps the sequence of Enh-II [9]. Hepatocyte-enriched transcription factors, such as hepatocyte nuclear factor1 (HNF1) [10], HNF3 [11, 12], HNF4 [13, 14], CCAAT/enhancer-binding protein (C/EBP) [15–17], and fetoprotein transcription factor (FTF) [18], which are known to be responsible for the hepatocyte-specific Enh-II activity, and regulate its function [19]. Different mutations in the Enh-II region (as T1653 mutations in box-α) have been identified which are responsible for the development of HCC. Most of the studies primarily focused on certain single point nucleotide mutations, however, it is unclear whether a single or specific combination of these mutations is associated with the development of HCC [20].

Pakistan is highly endemic for HBV with approximately nine million people infected and the infection rate is on a steady rise [21, 22]. Even though in Pakistan data related to HBV and its genotype prevalence are available but studies that cover information of HBV mutations especially in the Enh-II region and its clinical association with the development of HCC are scarce. This study is therefore designed to assess the diversity of mutations associated with LC and HCC in Enh-II regions in HBV positive patients of Khyber Pakhtunkhwa (KP) province, Pakistan.

## Materials and methods

### Study population

Samples were collected randomly from clinically confirmed HBV-positive patients from five districts (Peshawar, Mardan, Dir Lower, Kohat, Bannu) of KP, Pakistan.

### Samples and data collection

Blood (3mL) was collected in a sterile tube from HBs Ag-positive patient. Clinical data were obtained through a predesigned questionnaire. The samples were transported to the Laboratory of Virology and Immunology, Department of Zoology University of Peshawar for further process. Proper consent was obtained from each participant before including in the study and the study protocol was approved by the Research Ethical Committee, University of Peshawar, Pakistan.

### Inclusion/Exclusion criteria

Patients positive for HBsAg through Immunochromatography (ICT) test was included while those with HIV and HCV antibody positivity and whose HBV DNA was undetectable by PCR were excluded from the study.

### DNA extraction

HBV DNA was extracted by using DNA isolation kits (TRIzol TM Reagent, ThermoFisher scientific) as per the manufacturer’s instructions.

### HBV DNA and genotype/s detection

HBV DNA was detected through the protocol of Kyaw et al [23]. HBV genotype/s (A-F), were identified using the protocol of Naito et al [24]. Briefly, the first round of PCR was performed for the HBV S gene using, 10 pM of primers (P1 and S1-2) and green Master Mix having a final volume of 20 μL. The thermocycler conditions were adjusted to 10 minutes of denaturation at 95°C followed by amplification for 35 cycles (94°C for 20 sec, 55°C for 20 sec, and 72°C for 1 minute), and a final extension at 72°C for 7 minutes. Second-round PCR primers were mixed in two separate mixes as Mix-A and Mix-B depending on their targets and amplification size differences.

### HBV X gene amplification and detection

HBx gene was amplified through nested PCR using the protocol of Al-Qahtani et al [25]. Briefly, first-round PCR consisted of 10 μL of extracted DNA, 20 pM of each primer F1: 5′ ATTGATTGGAAAGTMTGTM 3′ and R1: 5′ TCCACAGTAGCTCCAAATTCTTT 3′ primer, and Green Master Mix (20 μL). The thermocycler conditions were initial 5 minutes of denaturation at 94°C followed by amplification for 30 cycles (94°C for 45 sec, 55°C for 30 sec, and 72°C for 1 minute), and a final extension at 72°C for 10 minutes. The second round of PCR primers were F2: 5′ CGCTTGTTTTGCTCGCAGC 3′ and R2: 5′ GGCACAGCTTGGAGGCTTG 3′ and the reaction and PCR conditions were the same as for the previous round.

### Gel electrophoresis

PCR amplified product was electrophoresed on 2% agarose gel, stained with ethidium bromide, and compared with 100bp DNA ladder (ThermoFisher USA). The amplified product was visualized under an ultraviolet (UV) trans-illuminator.

### Sequencing of DNA

Amplified HBV X gene was sequenced through Sanger method from commercially available services. The obtained sequences were submitted to GenBank with accession numbers, MZ666825, MZ676992, MZ676993, MZ676994, MZ676995, MZ711089, MZ711090, MZ711091, MZ711092.

### Trimming of enhancer II region

Redundant sequences present upstream and downstream of the Enh-II region were trimmed using BioEdit software and final sequences were obtained.

### Sequence analysis

Sequences were analysed for potent mutations using online sequence alignment tools like BLAST (https://blast.ncbi.nlm.nih.gov/BlastAlign.cgi). BioEdit software was used for the alignment of query sequences with the subject and different genotypes (A-J) sequences to compare similarities and differences.

### Phylogenetic analysis

Phylogenetic analysis was performed by comparing the obtained gene sequences with HBV reference sequences for each genotype available in Genbank. A phylogenetic tree was constructed in MEGA software (version 6.1) using the neighbor-joining method. Tamura Nei Model method was used to estimate the distances. Bootstrap statistical analysis was performed using 1000 datasets.

### Data analysis

Statistical software SPSS version 11.5, package (SPSS Inc, Chicago, IL, USA) was used for statistical analysis of the data. The data were compared by applying the student’s t-test and statistical significance was given to the groups having p-values of <0.05.

## Results

### Demographics of the study population

**Fig 1:**
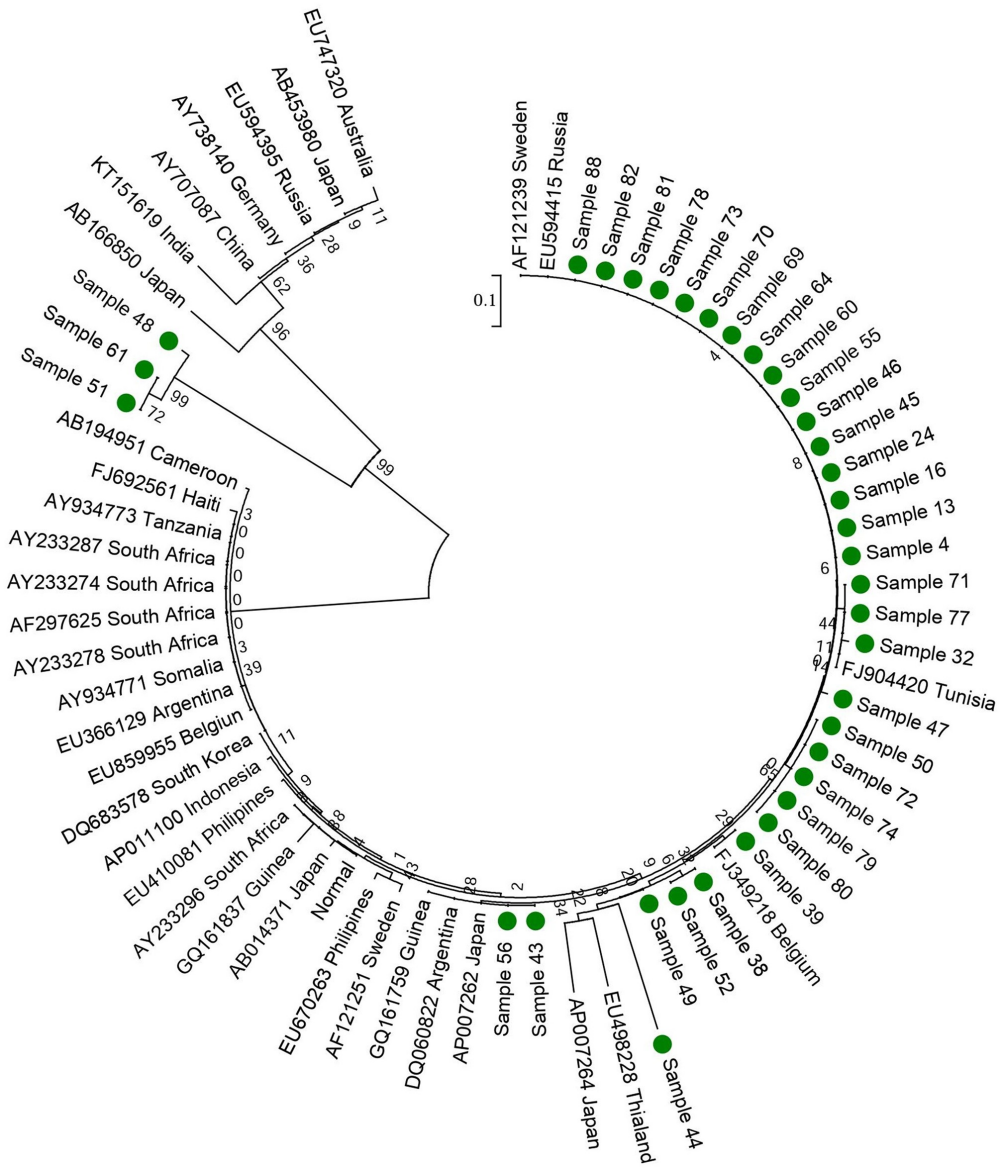
A rooted phylogenetic tree of 38 enhancer II region sequences obtained from Pakistani HBV patients aligned with reference sequences using neighbour joining method. Bootstrap statistical analysis was performed using 1000 datasets. The sequences are labelled by their lab number and country.

Different baseline characteristics such as age, sex, HBeAg status of these positive samples are summarized in Table 1. Phylogenetic analysis of HBV showed that all patients (100%) had genotype D (Fig 1). Among 200 HBsAg positive HBV patients, 148 had PCR confirmed HBV infection while 52 were PCR negative. Out of these 148 PCR positive samples, 125 (84.45%) had CH, 16 (10.81%) had HCC and 7 (4.72%) had LC. Gender wise study of HBV infection revealed a higher frequency of HCC in males (63.2%) patients as compared to females (36.8%) and was most prevalent in age group ≤40 years (60.8%), 40–49 years (30.4%), 50–59 years (17.6%) and >60 years (9.6%). The p-value was significant for all three groups (CH, LC, and HCC).

**Table 1:** General characteristics of HBV patients.

Basic characteristics	CH N (%)	HCC N (%)	LC N (%)	p-value
**Age in years**				
≤40	64 (51.2)	8 (50.0)	3 (42.8)	0.0238
40–49	31 (24.8)	5 (31.2)	2 (28.6)	
50–59	19 (15.2)	2 (12.5)	1 (14.3)	
≥60	11 (8.8)	1 (6.25)	1 (14.2)	
**Sex**				
**Male**	79 (63.2)	10 (62.5)	5 (71.4)	0.9041
**Female**	46 (36.8)	6 (37.5)	2 (28.6)	
**HBeAg**				
Positive	84 (67.2)	9 (56.2)	6 (85.7)	0.3785
Negative	41 (32.8)	7 (43.7)	1 (14.3)	

### Mutations in the Enh-II region

**Fig 2:**
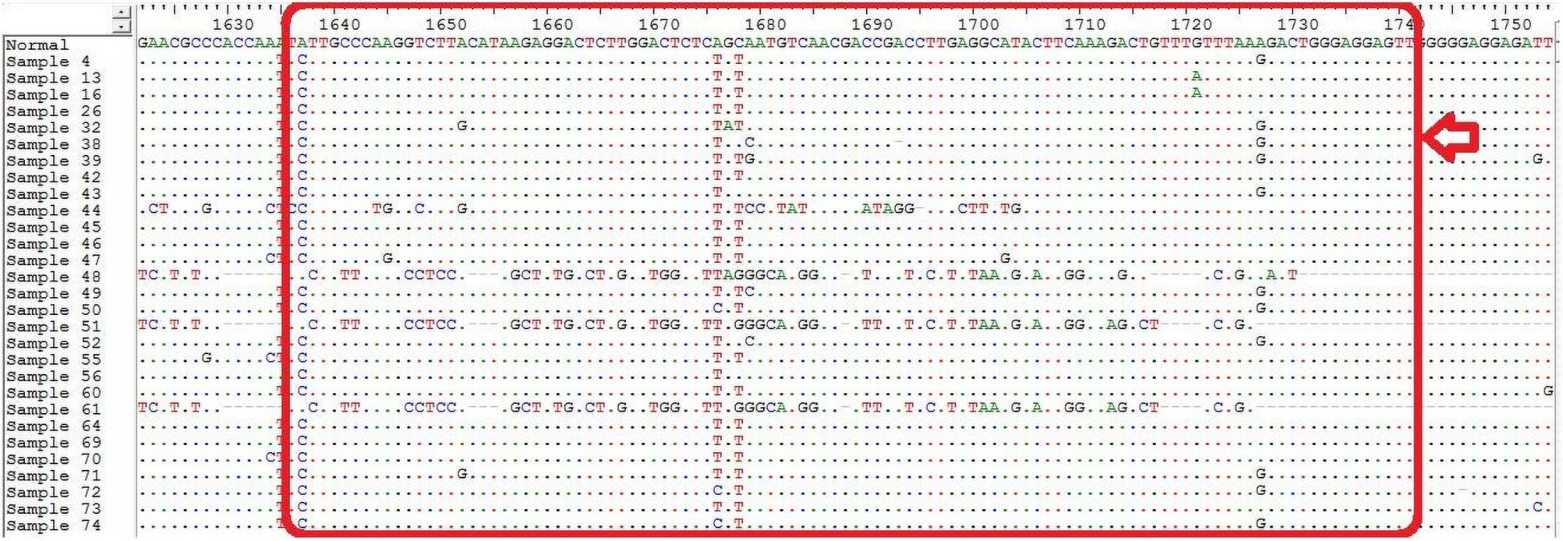
Enhancer II region nucleotide alignment showing comparison with the representative sequence (Normal). The dots represent similar nucleotides.

**Fig 3:**
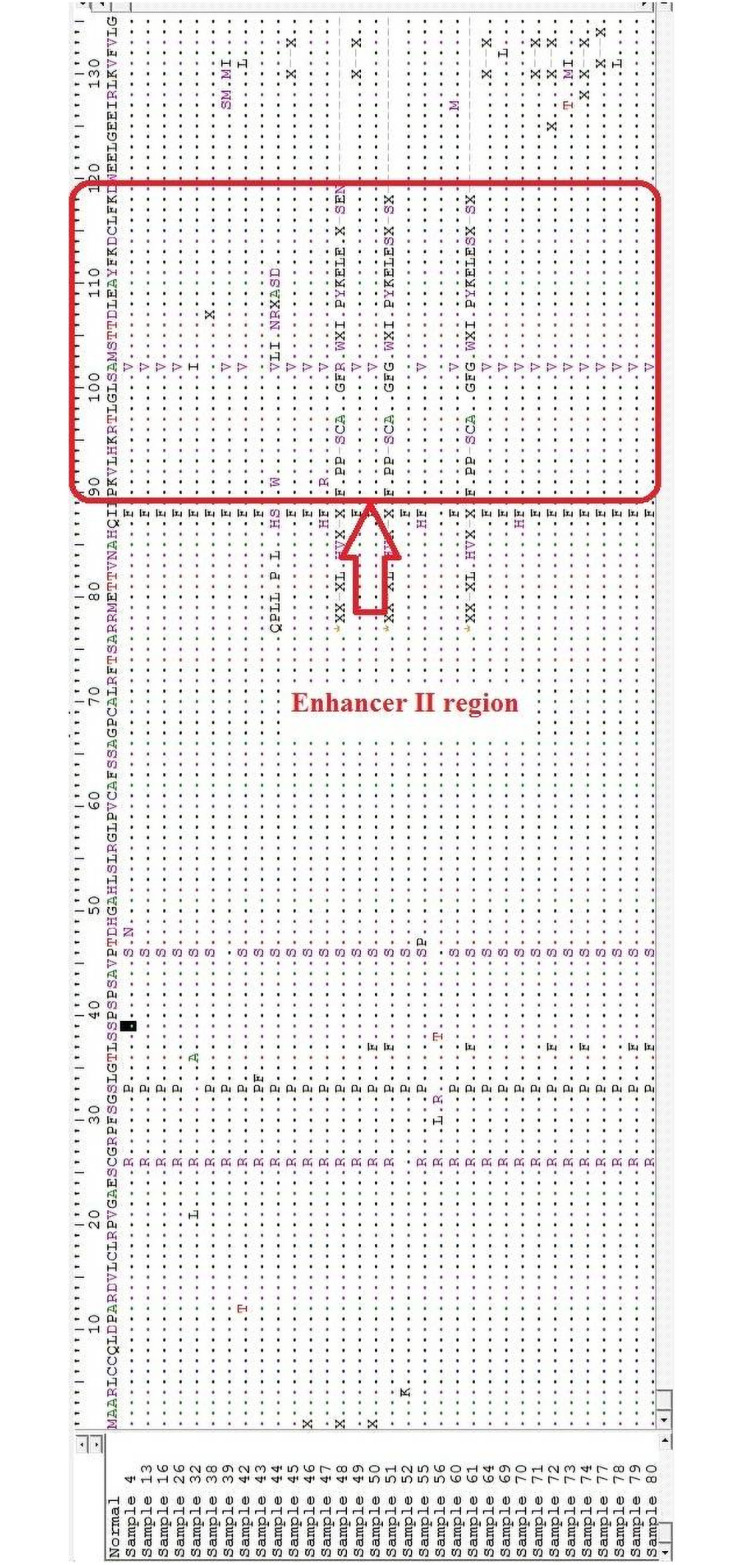
Comparison of the enhancer II region amino acid sequence with the reference sequence (Normal) showing amino acid substitutions and deletions.

Twenty-five different substitution and deletion mutations were observed in HBV-positive individuals in this study which are illustrated in Table 2. Sequencing and data analysis showed the highest frequency of S101F (62.2%), A102V/R/G/I (56.25%), and M103L/A (68.75%) mutation in HCC and 57.1%, 42.8%, and 28.52% respectively in LC as compared to 16%, 15.2%, and 18.4% respectively in CH patients. H94 deletion in the α-box of the Enh-II region has been reported to increase the risk of HCC and its frequency was higher in HCC (50%) as compared to and LC (28.5%) and CH group (6.5%) in this study. Deletion mutations of various sizes were also reported in this study. Moreover, some non-reported mutations such as E109A/Y (43.75%), A110S/K (37.5%), Y111D/E (50%), and F112L (43.75%) were also found in high frequency in HCC and LC (14.28%, 14.28%, 28.2% and 42.8% respectively) patients as compared to CHB (12.8%, 15.2%, 16.8% and 16% respectively) (Figs 2 and 3).

**Table 2:** Mutations in the Enh-II region (1636–1744).

Nucleotide						95% Conf. Interval	
	Amino Acid	CH N (%)	HCC N (%)	LC N (%)	*P* value	Lower	Upper
CCG1640TTT	P90F	12 (9.6)	4 (25)	1 (14.28)	0.0247	2.0911	19.869
AAA1644TGG/CGT	K91W/R	7 (5.6)	3 (18.75)	2 (28.57)	0.054	-0.2736	21.914
GTG1647GCG/CCG	V92A/P	11 (8.8)	5 (31.25)	1 (14.28)	0.0399	0.8127	22.964
T1650C	L93P	10 (8)	4 (25)	1 (14.28)	0.0307	1.4327	19.327
CAT1653Deletion	H94-	7 (6.5)	8 (50)	2 (28.5)	0.0758	-2.5629	36.563
AA1657GC	K95S	6 (4.8)	3 (18.75)	1 (14.28)	0.038	0.6521	15.291
CGT1659TGC	R96C	9 (7.2)	5 (31.25)	3 (42.85)	0.0597	-0.9819	33.749
ACC1662GCG	T97A	5 (4)	2 (12.5)	1 (14.28)	0.0363	0.6105	12.316
CT1670GG	L100G	16 (12.8)	8 (50)	2 (28.52)	0.0399	1.3328	37.774
AGC1674TTT	S101F	20 (16)	10 (62.2)	4 (57.1)	0.0396	1.9843	54.449
GCG1677GTG/ATT/CGT/GGC	A102V/R/G/I	19 (15.2)	9 (56.25)	3 (42.8)	0.0357	2.3877	46.029
ATG1680/CTG/GCG	M103L/A	23 (18.4)	11(68.75)	2 (28.52)	0.0446	0.8982	49.658
AGC1683ATT/TGG	S104I/W	20 (16)	4 (25)	1 (14.28)	0.0165	3.6732	23.087
CC1689AC/TT	T106N/I	17 (13.6)	7 (43.75)	2 (28.52)	0.0305	2.6013	34.689
GAT1692CGT	D107R	15 (12)	6 (37.5)	1 (14.28)	0.0386	1.1071	27.486
TG1695CG	L108P	14 (11.2)	5 (31.25)	2 (28.52)	0.0267	2.6423	28.014
GAA1699GCG/TAT	E109A/Y	16 (12.8)	7 (43.75)	1 (14.28)	0.0469	0.3171	31.293
GCG1701AGC/AAA	A110S/K	19 (15.2)	6 (37.5)	1 (14.28)	0.03	2.2361	28.757
TAT1704GAT/GAA	Y111D/E	21 (16.8)	8 (50)	2 (28.2)	0.0291	3.1875	38.812
TTT1707CTG	F112L	20 (16)	7 (43.75)	3 (42.8)	0.0271	3.7241	40.459
A1710G	K113E	17 (13.6)	6 (37.5)	2 (28.52)	0.0249	3.2839	31.589
AT1714GC	D114G	18 (14.4)	7 (43.7)	1 (14.28)	0.0413	0.9599	31.833
TTT1722AGC	F117S	21 (16.8)	5 (31.25)	2 (28.52)	0.0162	4.8376	30.019
AAA1725GAA/ATT	K118E/I	15 (12)	6 (37)	1 (14.28)	0.0375	1.2172	27.209
GAT1728AAC	D119N	22 (17.6)	5 (31.25)	2 (28.28)	0.0154	5.0792	30.297

### Mutation in transcription factors controlling Enh-II activity

**Fig 4:**
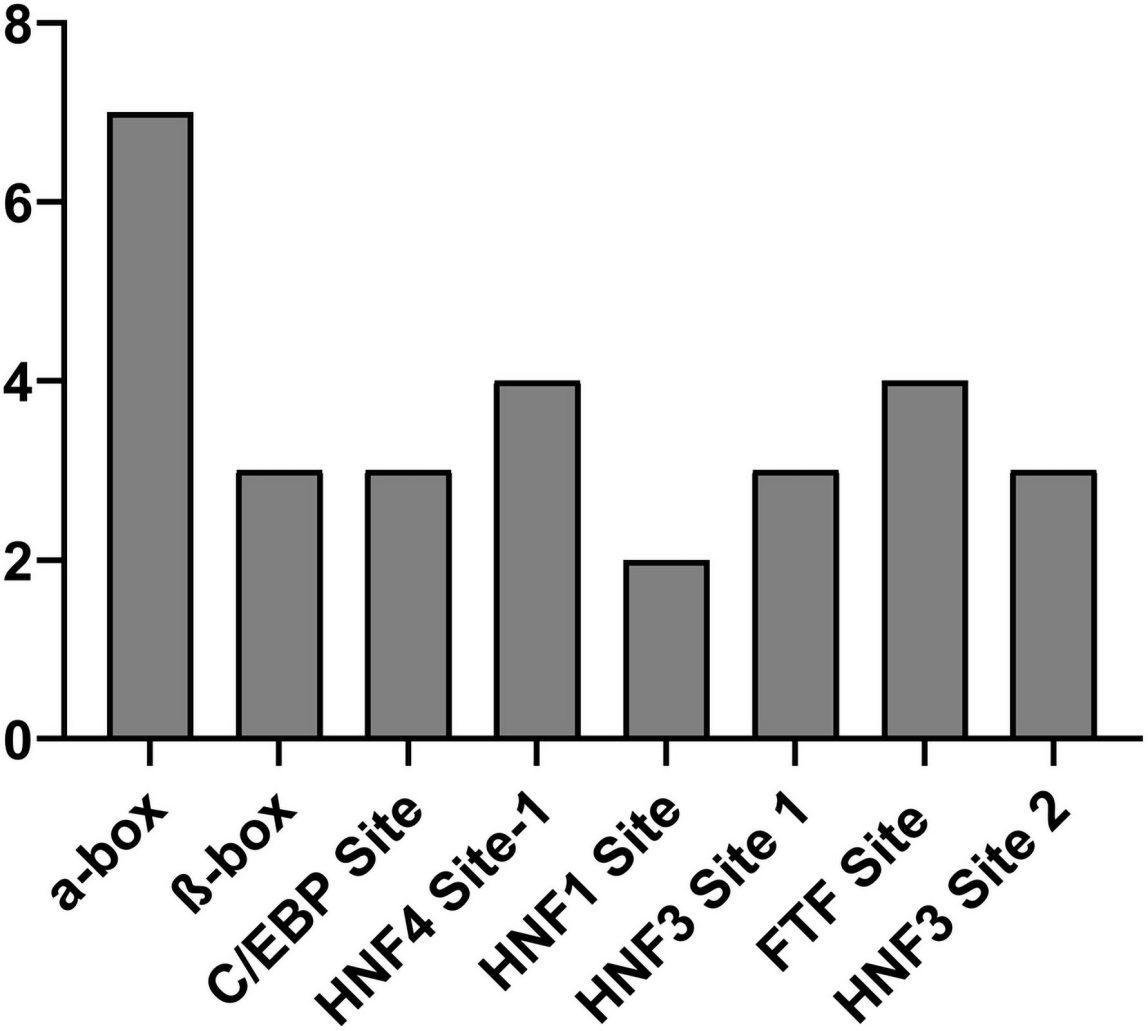
Frequency of mutation in different transcriptional factors controlling enhancer II activity.

**Fig 5:**
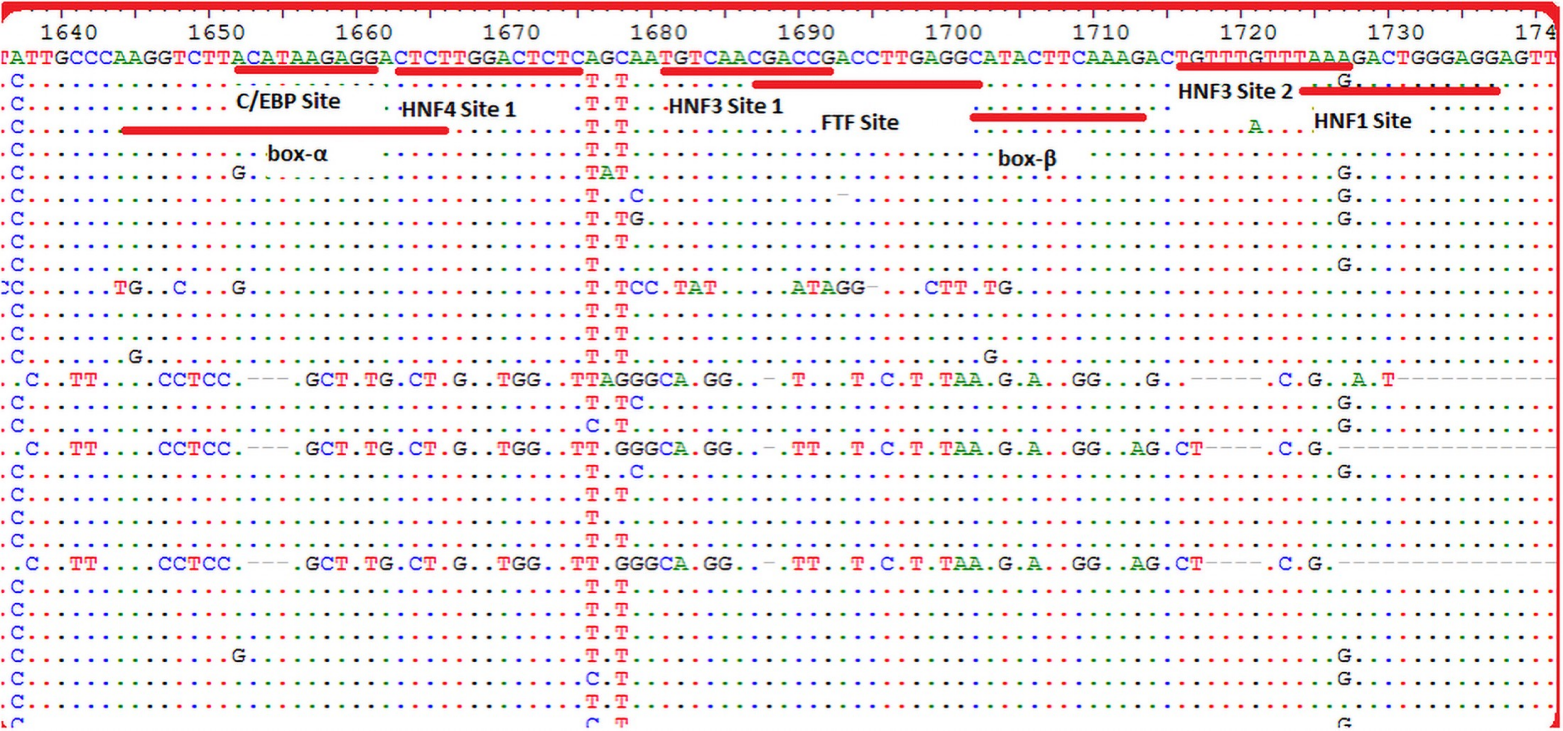
Mutations in different transcriptional factors affecting enhancer II activity.

In this study mutations in hepatocyte-enriched transcription factors HNF1, HNF3, HNF4, FTF (hB1F), C/EBP, α-box, and β-box were also found which are shown in Fig 4. The highest frequency of mutation was found in α-box followed by the HNF4 site 1 while the lowest frequency of mutation was observed in HNF1 site (Fig 5).

### Single, double and triple nucleotide(s) mutations causing amino acid substitution in CH, LC, and HCC patients

**Fig 6:**
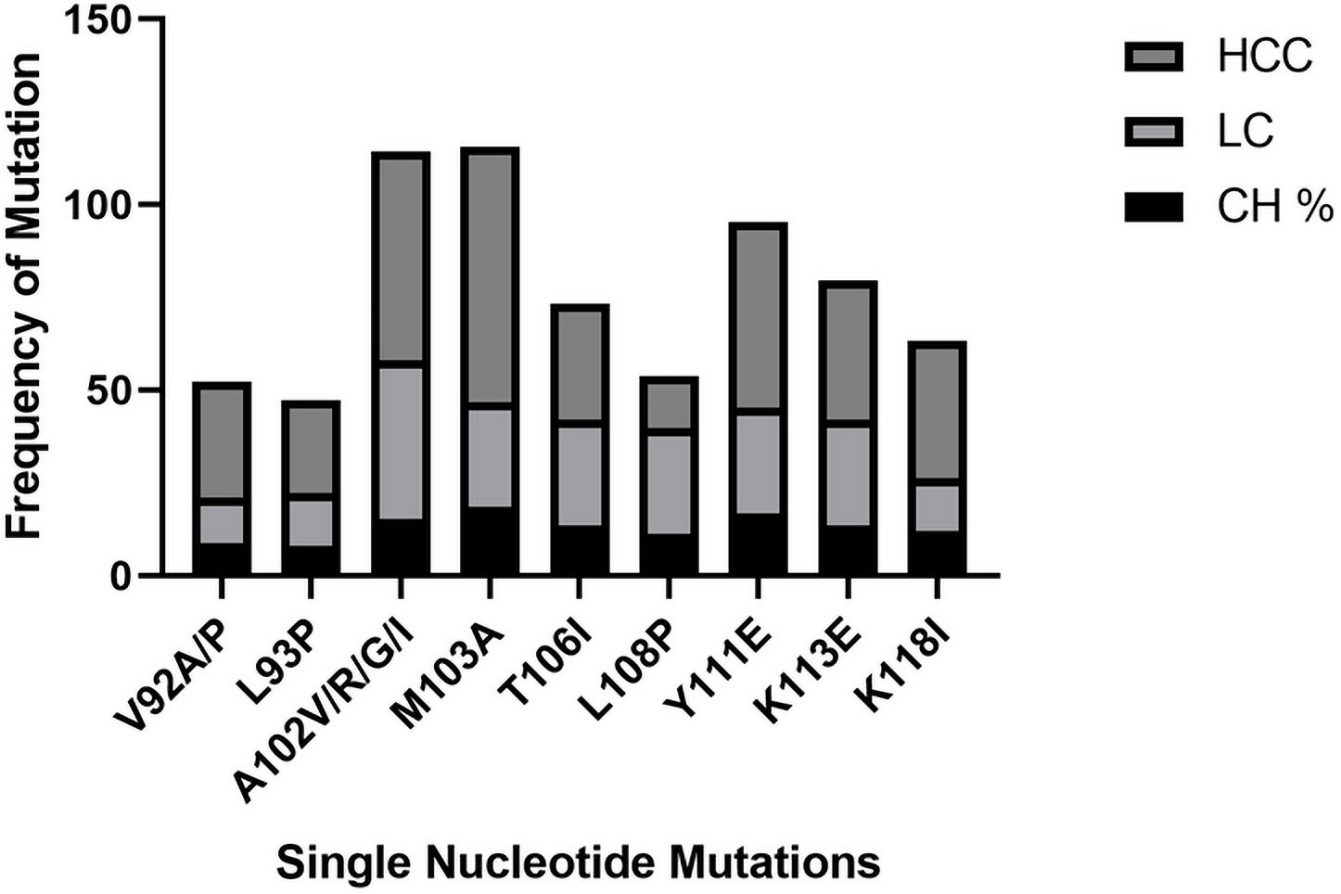
Frequency of single nucleotide mutations in CH, LC, and HCC groups.

**Fig 7:**
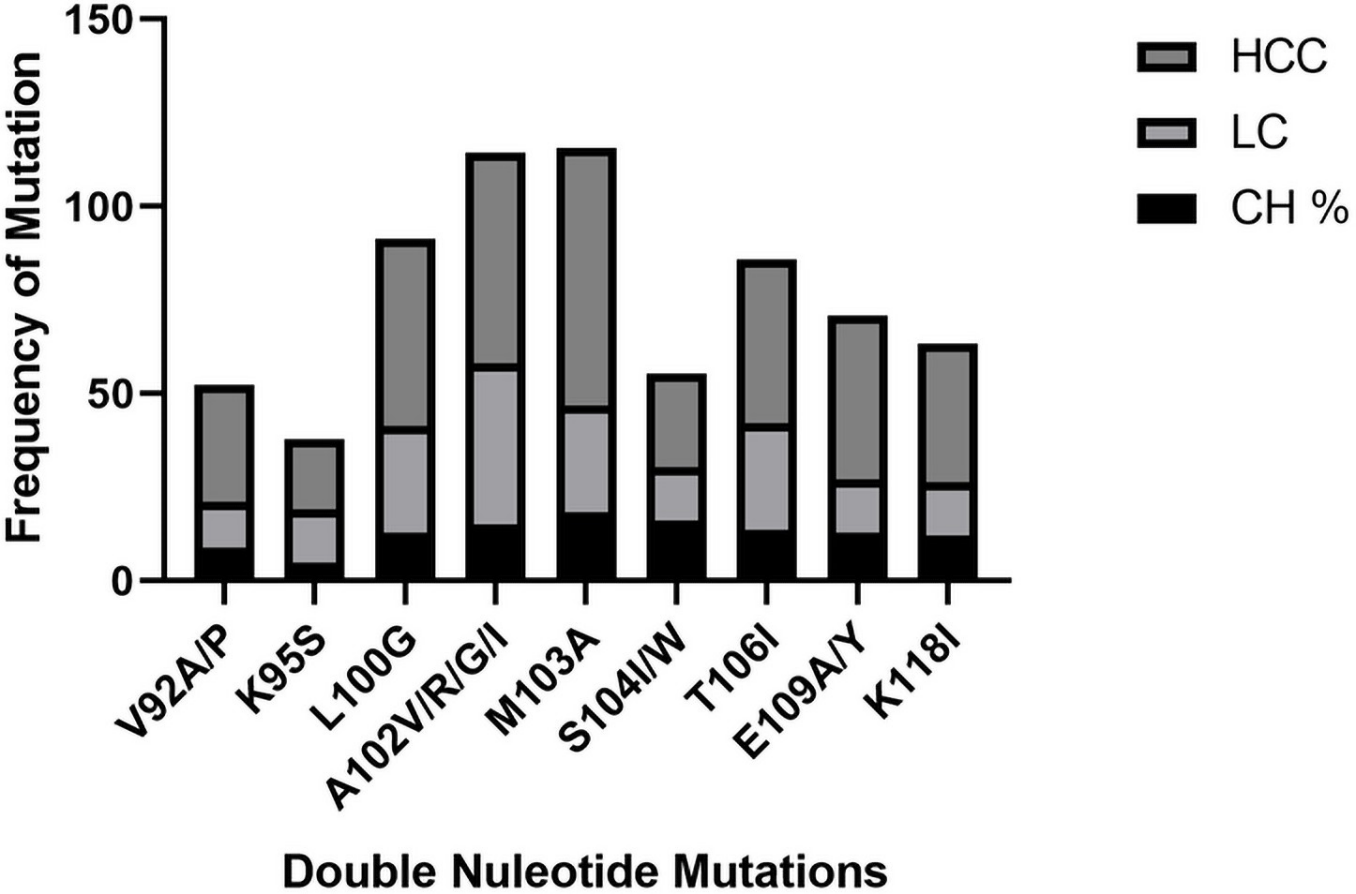
Frequency of double nucleotide mutations in CH, LC, and HCC groups.

**Fig 8:**
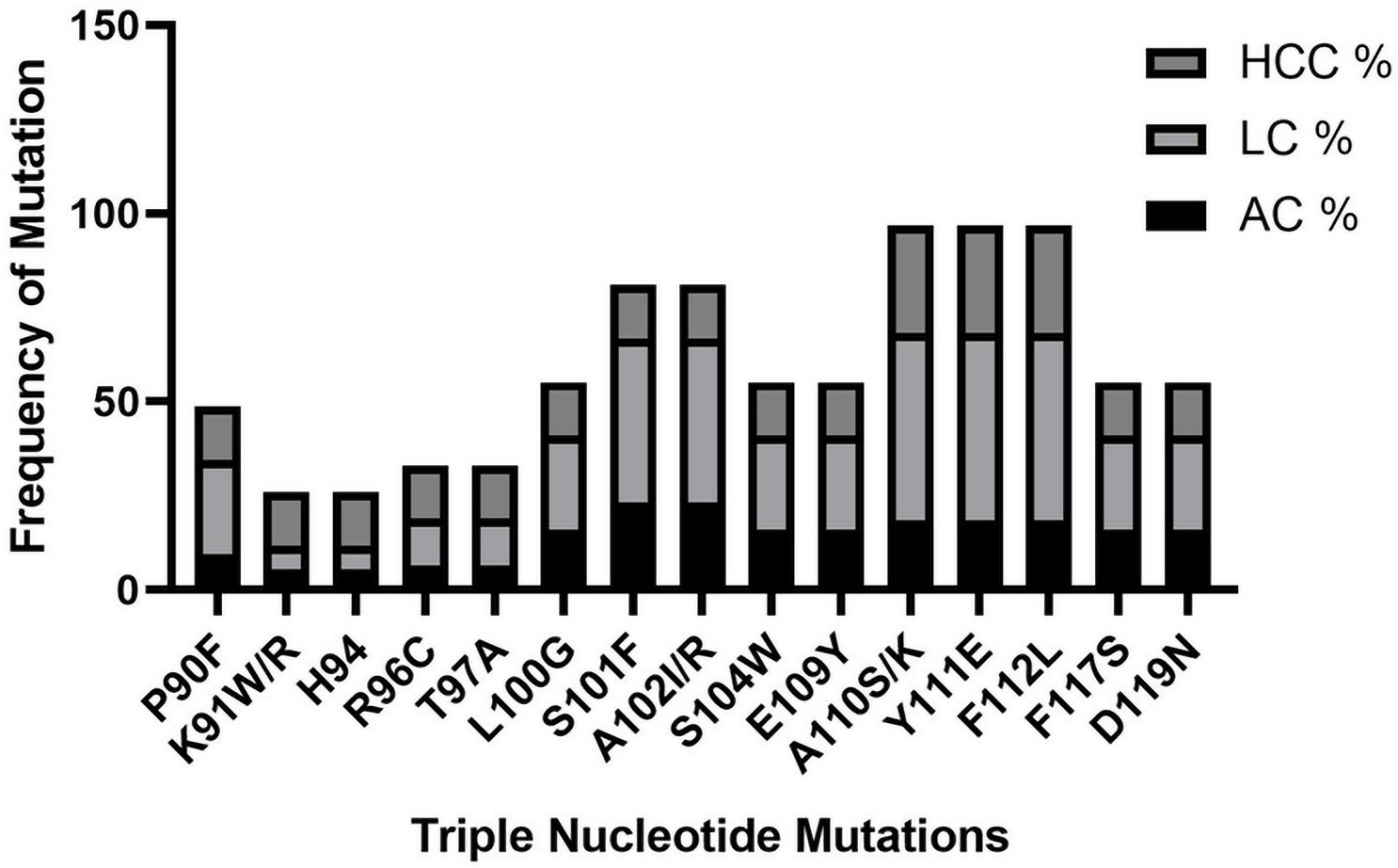
Frequency of triple nucleotide mutations in CH, LC, and HCC groups.

The significance of mutations related to HCC progression was evaluated by Univariate and multivariate logistic regression analysis among CH, LC, and HCC groups. Nine single nucleotide mutations were found in the Enh-II region, including the highest incidence rate of mutation L108D, Y111D, K113E in HCC as compared to other groups. Similarly, 9 double nucleotide mutations were also detected in different regions of the Enh-II region having A102G, M103A, and K118I in HCC compared to CH and LC. Fourteen triple nucleotide mutations were found among which the more frequent mutation in LC and HCC groups were A110S/K, Y111E, and F112L while K91W/R and R96C were found in the lowest frequency (Figs 6–8).

### Deletion or insertion mutations in Enh-II region

We found 17 different patterns of deletion. The deletion was observed in different nucleotides of the Enh-II region such as 1687, 1688, 1693, and 1695. Similarly, nt deletions 1718→1721 (23.64%) and 1630→1635 (18.24%) were found as the most frequent deletion mutations. A nucleotide deletion of 5 bp was detected in 16.86% (1610→1614) and 3.37% (1717→1721) cases while 6 nt deletion was found at 1630→1635 in 18.24% cases with a remarkable pattern. Two different nt insertions in the X gene were CCTCCA (6 bp) and AGGGCA (6 bp) at nt 1647 and nt.1677 were also found. We also found truncation in the Enh-II region from 1726→end in 10.81% cases and 1731→end in 14.84% cases (Table 3).

**Table 3:** Deletion mutation in the Enh-II region of HBV.

Length of Fragment	Nucleotide Deletion	Frequency	Percentage (%)
**1 bp**	1687,	8	5.40
	1688,	4	2.70
	1693	11	7.43
	1695	17	11.48
	1746	4	2.70
	1757	17	11.48
**3bp**	1653→1655,	23	15.54
**4 bp**	1718→1721	35	23.64
**5 bp**	1610→1614,	22	16.86
	1717→1721	5	3.37
**6 bp**	1630→1635	27	18.24
**Truncated**	1731→end	16	10.81
	1726→end	22	14.84

### Non-reported mutations

We also found some non-reported mutations. Non-reported mutations such as E109A/Y (43.75%), A110S/K (37.5%), Y111D/E (50%), F112L (43.75%) were found in high frequency in the HCC group as compared to other groups (Table 2). Overall frequencies of non-reported mutations are given in Table 4.

**Table 4:** Non-reported mutations.

Nucleotide Mutation	Amino Acid Mutation	Frequency (N)	Percentage (%)	95% Conf. Interval		
				P values	Lower	Upper
T1650C	L93P	15	10.13	0.1218	−31.84	75.4
CAT1653 Deletion	H94-	17	11.48	0.1218	−31.84	75.4
CGT1659TGC	R96C	17	11.48	0.1218	−29.386	69.596
AGC1683ATT/TGG	S104I/W	25	16.89	0.1217	−36.681	86.951
GAA1699GCG/TAT	E109A/Y	24	16.21	0.1219	−20.829	49.309
GCG1701AGC/AAA	A110S/K	26	17.56	0.1219	−18.375	43.505
TAT1704GAT/GAA	Y111D/E	31	20.94	0.1219	−20.829	49.309
TTT1707CTG	F112L	30	20.27	0.1217	−30.579	72.469
AT1714GC	D114G	26	17.56	0.1218	−37.942	89.882

## Discussion

HBV infection is one of the leading causes of the advanced form of liver disease and continues to remain a medical concern in Pakistan despite prophylaxes. Most of the studies in Pakistan have been focussing on HBV’s prevalence, its epidemiology, and genotyping [22]. Studies are scarce on HBV’s mutations and their association with advanced forms of liver disease. Previously, the association of mutations in Enh-II region with the development of advanced liver disease were not investigated in this area. Various studies around the globe suggested that certain mutations in the HBV genome may be associated with HCC development [26, 27]. It is considered that mutations in HBV genome may responsible for escape from the host immune system and may affect the oncogenic potential inchronic HBV diseases [28]. Sequences in this study revealed nucleotide substitutions, deletions, and insertions within the Enh-II region, which are shown associated with certain biological functions of the protein that may lead to cancer.

Deletion mutation was found in this study at nt 1653 (H94-) in 6.5% of CH, 28.5% of LC, and 50% of HCC patients which showed an increasing trend from CH to HCC. These mutations might be a potential biomarker to predict the prognosis for patients with an advanced form of liver diseases such as LC and HCC. Our findings are consistent with other studies [20, 29] thatreported high frequency of HCC due to substitution mutation at nt 1653 in the Enh-II region of the HBx gene. But in our study at this point, it was a deletion mutation. This region in Enh-II is located in the box α which is a strong activation element of the Enh-II/core promoter that can enhance the box α binding affinity and Enh-II/core promoter activity [15, 29]. Many trans-regulated nuclear factors bind HBV at the 1653 site, and such mutation can alter the binding affinity of the nuclear factors [30, 31]. Mutation at this site was reported to be a predictive factor for HCC in genotype C [20] and genotype D [32]. A meta-analysis data also resported that C1653T is associated with an increased risk of HCC [33].

Substitution mutation S101F was detected in a considerable number of samples (N = 37) and found highest in HCC patients (62%) followed by LC patients (57%). It is shown that mutation at this site is responsible for the disturbance of the cell cycle leading to apoptosis caused by the increased expression of p21 in Hep G2 and may lead to the advanced form of liver disease [4]. The level of HBV transcription varies with the extent of transcription factor binding to HBV DNA, which can be altered by mutations in the HBV genome. When a new mutation allows a transcription factor to bind a new region in HBV DNA, HBV replication is stimulated resulting in the progression of the disease [34]. The substitution mutation T106N/I associated with HCC [35] was found in 43.75% of HCC and 28.52% ofLC patients of this study. The Enh-II region from nt 1640 to 1663 containing box-α and C/EBP site is an independent sequence and sufficient for the enhancer activity. In an experimental study, mutation in this sequence results in a great decrease in luciferase gene expression under the control of the minimal promoter [10]. In the present study eight different (P90F, K91W/R, V92A/P, L93P, H94-, K95S, R96C, T97A) substitutions and deletion mutations were found in this region that may affect the viral replication and cell cycle and might play a role in the development of the advanced form of liver disease.

Hepatocyte enriched transcription factors have various binding motif and regulate the activities of Enh-II region [10–17]. Of these transcriptions factors, HNFs regulate cccDNA transcription by directly interacting with HBx, which enhances the DNA-binding activity of HNFs. HNF1 up-regulates Enh-II activity by interacting with either the hB1F or B element in Enh-II. In the absence of HNF1α, the concentration of HBV pgRNA is decreased, resulting in decreased genome replication [31]. In this study, two mutations were found in the HNF1 Site (K118E/I and D119N) which were more prevalent in HCC (37%) patients as compared to LC (14.2%) and CH (12%). It is reported that due to mutations new binding sequences ofHNF1 may emerge that activate increased viral replication. Moreover, double combinational mutation (K118E/I, D119N) within regulatory elements of Enh-II has been shown to affect the replication of HBcAg in situ and can manifest liver disease [36, 37]. Additionally, this double mutation was studied in T cellular cell epitope (amino acid 115–123) and T helper cell epitopes (amino acid 111–135) which plays a role in the evasion of the HBV from host immune clearance [38]. In the current study, 4 bp deletion was found in the SP1 binding site of the C-terminal region (nt 1717–1721). Deletion in this region causes carcinogenesis which might be due to the deletion of the Sp1 binding site that controls the expression of the X gene negatively. The deletion of the Sp1 binding site is increased with the expression of the X gene [39].

Likewise, three mutations T97A, L100G, and S101F were reported in the HNF4 site which were more prevalent in HCC groups (12.5%, 50%, and 62% respectively) as compared to other two groups. Such deletion may increase HNF4 expression and HNF3 down-regulation in CH patients [35].

Overall, twelve non-reported mutations were also identified (P90F, V92W/R, R96C, T97A, S104I/W, E109A/Y, D107R, A110S/K, Y111E, F112L, and D114G). Mutations in the Enh-II region of the amino-terminal domain of core protein may be responsible for the nucleocapsid assembly and stability [40]. The incidence and impact of these mutations are unclear; however, the presence of these mutations in high frequency in HCC and LC patients is alarming as these mutations might be responsible for the advanced form of liver disease.

## Conclusion

In conclusion, in this age, sex, and HBeAg status matched study, most of the mutations were associated with HCC development in infected patients of KP with HBV/D which are also confirmed by other studies. The occurrence of multiple mutations represents a strategy by which HBV can escape immune surveillance and thus contribute to hepatocarcinogenesis. These mutations cause structural changes and conformation alteration of X protein and hence alter the regulatory and transactivation functions. Some unreported mutations were also detected in our study which may provide new insights into the mechanism by which multi-site mutations in the Enh-II region may contribute to the development of advanced liver disease. However, further experiments need to be conducted to confirm the inference. In short, our study confirms certain mutations and their association with an advanced form of liver disease in Pakistani patients.
